# Surprisingly little population genetic structure in a fungus-associated beetle despite its exploitation of multiple hosts

**DOI:** 10.1002/ece3.560

**Published:** 2013-04-17

**Authors:** Corlett W Wood, Hannah M Donald, Vincent A Formica, Edmund D Brodie

**Affiliations:** 1 Mountain Lake Biological Station, Department of Biology, University of VirginiaCharlottesville, Virginia, 22904; 2 Department of Biology, Swarthmore CollegeSwarthmore, Pennsylvania, 19801

**Keywords:** *Bolitotherus cornutus*, community diversity, environmental heterogeneity, resource-associated population structure

## Abstract

In heterogeneous environments, landscape features directly affect the structure of genetic variation among populations by functioning as barriers to gene flow. Resource-associated population genetic structure, in which populations that use different resources (e.g., host plants) are genetically distinct, is a well-studied example of how environmental heterogeneity structures populations. However, the pattern that emerges in a given landscape should depend on its particular combination of resources. If resources constitute barriers to gene flow, population differentiation should be lowest in homogeneous landscapes, and highest where resources exist in equal proportions. In this study, we tested whether host community diversity affects population genetic structure in a beetle (*Bolitotherus cornutus*) that exploits three sympatric host fungi. We collected *B. cornutus* from plots containing the three host fungi in different proportions and quantified population genetic structure in each plot using a panel of microsatellite loci. We found no relationship between host community diversity and population differentiation in this species; however, we also found no evidence of resource-associated differentiation, suggesting that host fungi are not substantial barriers to gene flow. Moreover, we detected no genetic differentiation among *B. cornutus* populations separated by several kilometers, even though a previous study demonstrated moderate genetic structure on the scale of a few hundred meters. Although we found no effect of community diversity on population genetic structure in this study, the role of host communities in the structuring of genetic variation in heterogeneous landscapes should be further explored in a species that exhibits resource-associated population genetic structure.

## Introduction

Most natural populations exist in spatially heterogeneous environments. Because environmental heterogeneity impacts two forces of evolution – selection and gene flow – it often shapes patterns of genetic variation at the landscape level (Manel et al. 2003; Hanski et al. 2011). Environmental features as diverse as physical impediments (e.g., mountain ranges, Rueness et al. 2003; waterfalls, Castric et al. 2001), habitat fragmentation (Haag et al. 2010), and microhabitat variation (Stireman et al. 2005; Cano et al. 2008; Ferrari et al. 2008) all function as barriers to gene flow, producing patterns of population genetic structure (hereafter, “population structure”) that are coincident with the landscape. Understanding when and how the landscape influences population structure is essential to delineate the circumstances that constrain and foster phenotypic evolution and local adaptation.

One such pattern typically found in heterogeneous environments is resource-associated population structure, in which populations exploiting distinct resources are genetically differentiated (Stireman et al. 2005; Ferrari et al. 2012). It generally results from divergent selection in different environments, which selects against migrants, and habitat choice, which limits migration between environments (Kawecki and Ebert 2004; Ficetola and Bonin 2011). Resource-associated population structure is well documented in natural populations and demonstrates how the environment can impact gene flow, even in the absence of physical barriers (Feder et al. 1994; Mopper 1996).

Most studies, however, stop short of investigating how resource community diversity on the landscape level affects patterns of population structure. The pattern of resource-associated population structure that emerges in a given landscape should depend on its particular combination of resources; a population in an environment composed of a single resource should be less structured than one found in a landscape in which multiple resources are equally abundant. Furthermore, the cost of dispersal may be higher – and therefore dispersal less frequent – in heterogeneous environments because only a subset of encountered resources will be suitable. These effects of resource community composition on population genetic structure can be detected by measuring population genetic differentiation in communities that differ in the degree of resource heterogeneity. In a highly heterogeneous resource community, differentiation should be high because most pairwise comparisons will involve populations inhabiting different resources, between which gene flow is infrequent. By contrast, in a homogenous environment, all pairwise comparisons will be between populations on the same resource, between which gene flow is uninhibited, and population differentiation will be low.

Insects that exploit multiple sympatric host species are good systems in which to explore this question because the relative abundance of the hosts is spatially heterogeneous, creating a landscape that varies in terms of resource community structure. This heterogeneity of host availability may impact patterns of genetic structure in associated insects due to differential host preferences, as well as host-specific selection regimes (Resetarits 1996; Nosil and Crespi 2004; Refsnider and Janzen 2010).

**Figure 1:**
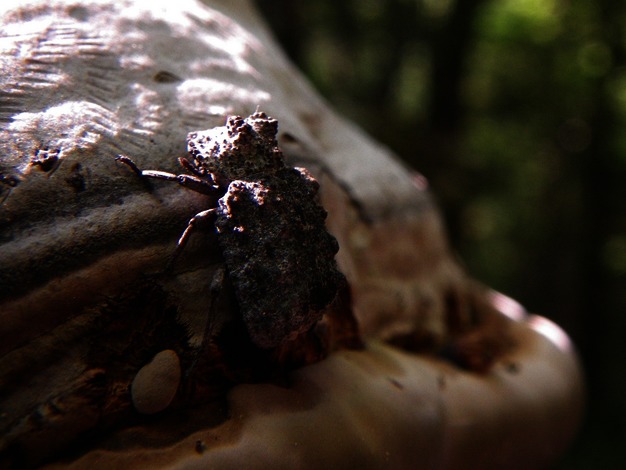
A female forked fungus beetle (*Bolitotherus cornutus*) on a fruiting body of *Fomes fomentarius*. Photo by V. A. Formica.

**Figure 2:**
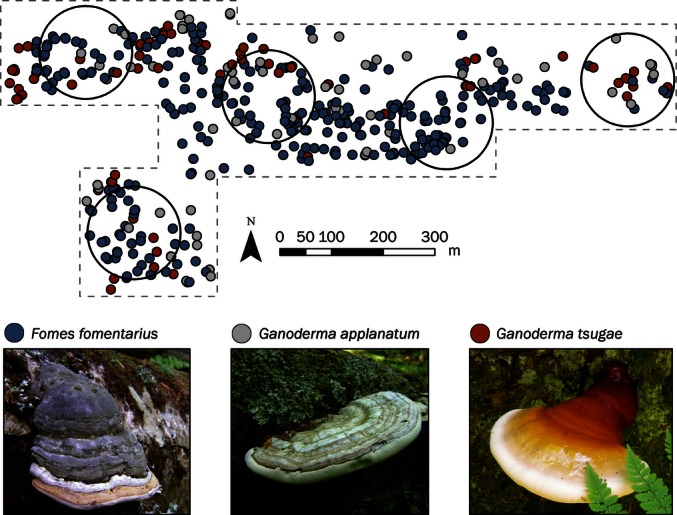
The distribution of the three host fungi in the landscape near Mountain Lake Biological Station in Giles County, VA. Top: Map of the study area. The circles delineate the five study plots, and the dotted lines indicate the area surveyed. Bottom: a fruiting body of each of the three fungi. Photos by C. W. Wood.

Here, we investigate the relationship between host community diversity and population structure in forked fungus beetles (*Bolitotherus cornutus*; Tenebrionidae; Fig. 1). These beetles rely on three species of polypore fungi (*Fomes fomentarius, Ganoderma applanatum*, and *Ganoderma tsugae*) that are sympatric over much of their range, and are often found growing within a few meters of each other (Gilbertson and Ryvarden 1986; Fig. 2). Host community diversity varies on a small spatial scale, allowing us to address the impact of the host community on population structure in a single metapopulation. A previous study conducted at approximately the same scale and location as this study, but only examining a single host (G. *applanatum*), found a moderate level of genetic differentiation (*F*_ST_ = 0.017; Whitlock 1992). This suggests that population structure exists on a microgeographic scale in *B. cornutus*.

Several lines of evidence suggest that the host fungi may contribute to population differentiation in *B. cornutus*. First, throughout their life cycle these beetles rely on live bracket fungi, which are probably well defended by secondary chemical compounds (Liles 1956; Jonsell and Nordlander 2004). Coevolution between insects and the chemical defenses of their hosts is common, and tends to drive specialization on a single host (Cornell and Hawkins 2003). Second, defensive volatiles differ among beetles collected from different host fungi, suggesting that individual beetles tend to associate with a single fungus type and that the fungi are characterized by distinct chemical compositions (Holliday et al. 2009). Moreover, endophagous insects like *B. cornutus*, whose larvae develop and pupate inside the brackets (Liles 1956), disproportionately exhibit local adaptation to a single host (Mopper 1996; Stireman et al. 2005). Third, the fungi themselves grow on different trees and have contrasting life histories, so their nutrient profiles may differ (Gilbertson and Ryvarden 1986). *F. fomentarius* and *G. applanatum* produce perennial brackets and grow mostly on hardwoods, whereas *G. tsugae* produces brackets annually and specializes on hemlock (*Tsuga* spp.; Brown and Rockwood 1986). Finally, experimental evidence suggests that *B. cornutus* discriminate among the three host fungi. In lab-based choice experiments, *B. cornutus* preferred to eat *G. tsugae*, and the same experiment suggested that females might prefer to oviposit on *G. applanatum* (Heatwole and Heatwole 1968). Discrimination among fungi is evident in the field as well. *B. cornutus* that originate on *G. tsugae* tended to disperse to other *G. tsugae*-infected logs (Schwarz 2006).

Here, we assess the effect of a heterogeneous host community on *B. cornutus* population structure on a microgeographic scale: an Appalachian forest in which all three host fungi are intermixed. We address two questions. First, does host community diversity affect the degree of genetic differentiation among *B. cornutus* subpopulations? We predict that in a community with low host species diversity, gene flow will be relatively unrestricted and genetic differentiation low. By contrast, gene flow will be more restricted and genetic differentiation high in a community in which all three hosts are equally abundant. The expected effect of host community diversity on population structure is based on the assumption that fungi contribute to population structure through resource-associated differentiation. To directly test for resource-associated differentiation, we ask whether host fungus contributes to population structure in *B. cornutus*, which would suggest that divergent selection and/or habitat choice may be acting as barriers to gene flow between beetle populations on different host fungi.

## Methods

### Study species

Adult *B. cornutus* feed, mate, and lay eggs on the fruiting bodies (“brackets”; Liles 1956) of the host fungi, and larvae burrow into the brackets and consume fungal tissue throughout development (Fig. 1). A single subpopulation consists of all individuals inhabiting a single log and the associated fungal fruiting bodies. Approximately 25–30% of individuals migrate to other logs during their life span (Whitlock 1992; Ludwig 2008), although these estimates were obtained in studies of subpopulations inhabiting only one of the main host fungi (*G. applanatum*). The average adult lifespan of *B. cornutus* is 69 days (Conner 1988), although some individuals live for several years (Pace 1967).

### Field sampling and genotyping

The study site was located on Salt Pond Mountain near Mountain Lake Biological Station in Giles County, Virginia. In May–June 2011, we surveyed an area of approximately 0.25 km^2^ (250 m × 1000 m) along two drainages, Hunters Branch and Pond Drain (37.373758 N, 80.535067 W), for the three host fungi (Fig. 2). We georeferenced all logs infected with *F. fomentarius*, *G. applanatum*, and *G. tsugae* with a handheld global positioning system (Garmin, Kansas City, KS).

Within the surveyed area we defined five circular study plots (Fig. 2) using geographic information systems software (ArcGIS, ESRI, Redlands, CA). Each of the plots had a radius of 90 m, a distance chosen because 75% of beetles disperse fewer than 90 m (Ludwig 2008). Plots were separated by 180 m. In June–July 2011, we visited each plot once per week for 3 weeks, and during each visit we searched all infected logs for *B. cornutus* adults. All *B. cornutus* were collected and brought back to the lab. Each individual received a unique ID, which was painted on its elytra using Testors Gloss Enamel in earth tone colors. We collected 0.2–5 μL of hemolymph from the defensive glands of all beetles using the method described in Donald et al. (2012), which did not affect either survival or reproduction in laboratory trials. Hemolymph was stored in prepared lysis buffer (Promega DNA IQ system). All beetles were returned to their location of capture within 72 h.

DNA extractions were performed with Promega's DNA IQ System (Promega 2010), and PCR was performed using Qiagen's Multiplex PCR kit and microsatellite protocol (Qiagen 2010). Fragment analysis was completed by GeneWiz Inc. (South Plainfield, NJ) using Applied Biosystems 3730xl DNA Analyzers. All individuals' genotypes at nine microsatellite loci (Donald et al. 2012) were scored using GeneMarker (SoftGenetics, State College, PA).

### Statistical analysis

#### Population genetic structure

We used hierarchical analysis of molecular variance (AMOVA) to test whether host fungi contribute to population structure in this system. Population differentiation was assessed using *F*-statistics, which rely on allele identity information (Weir and Cockerham 1984), rather than with *R*-statistics, analogs to *F*-statistics that are based on a stepwise mutation model and are often used for microsatellites (Slatkin 1995). To determine whether *F*- or *R*-statistics were appropriate for our data set, we performed a test of mutation effect on genetic structure in SPAGeDi 1.3 (Hardy and Vekemans 2002) based on 10,000 permutations (Hardy et al. 2003; Galligan et al. 2012). Because there was no significant effect of mutation on genetic structure in our data set (*P* = 0.483, *N* = 508 individuals), we performed all tests using *F*-statistics.

AMOVA partitions total genetic variance into covariance components that describe the correlation between random haplotypes within the same subgroup, relative to a larger group. For our analysis, individuals were nested within logs (subpopulations), and logs were nested within fungus species. On the basis of these hierarchical groupings, we calculated the following *F*-statistics: *F*_IS_, the correlation between haplotypes within individuals relative to their subpopulation; *F*_SC_, the correlation between haplotypes within subpopulations relative to all individuals from the same host fungus; *F*_CT_, the correlation between haplotypes within host fungi relative to the entire metapopulation; and *F*_ST_, the correlation between haplotypes within subpopulations relative to the entire metapopulation. A significant value for any of these *F*-statistics indicates significant population genetic structure with respect to the relevant group (i.e., a significant *F*_ST_ indicates genetic structure among subpopulations; a significant *F*_CT_ indicates significant genetic structure among host fungi).

All genetic analyses were performed using Arlequin (Excoffier et al. 2005), and significance testing was conducted using 10,000 permutations. A subpopulation was only included in these analyses if it had at least five genotyped individuals. We performed locus-by-locus AMOVA because some loci had missing data, and we are presenting the results for each of the nine microsatellite loci separately, as well as the weighted average over all loci. 95% confidence intervals for the multilocus *F*-statistics were obtained by bootstrapping over loci.

Because some logs were coinfected by more than one species of fungus (*N* = 9; 20.9% of all logs), we included a coinfected category as a fourth level of the fungus factor (*G. applanatum*, *G. tsugae*, *F. fomentarius*, and coinfected logs) in the AMOVA analysis. All but one of the coinfected logs were infected by *G. applanatum* and *F. fomentarius*; the remaining coinfected log hosted *G. applanatum* and *G. tsugae*. Excluding coinfected logs did not qualitatively alter our results.

Before performing the combined analysis, we ran a separate AMOVA to test for a significant effect of plot. Plot did not contribute significantly to total genetic variance (*P* = 0.91), allowing us to pool individuals across plots to test forest-wide structure. Finally, because sex-biased dispersal may result in sex-specific patterns of population structure, we also ran the above AMOVA separately for females and males. Only populations with at least five females or five males, respectively, were included in this analysis.

#### Isolation-by-distance

Because we did not detect population structure in the above analyses (see Results), we tested for isolation-by-distance on a larger geographic scale by augmenting our data set with 88 individuals collected from two sites 6 km (37.42196912 N, −80.50314923 W) and 9 km (37.45938702 N, −80.53336415 W) away from our primary study site on Salt Pond Mountain. At each of these sites, beetles were collected from several logs that were infected by either *F. fomentarius* or *G. applanatum* (6-km site: 9 logs; 9-km site: 11 logs). Because the host fungi did not affect population structure in the Salt Pond Mountain data set, these beetles were pooled by site for the isolation-by-distance analysis. Pairwise genetic distances (Slatkin's linearized *F*_ST_, D = *F*_ST_/(1-*F*_ST_)) were calculated using Arlequin, and pairwise geographic distances were calculated using the Geographic Distance Matrix Generator v 1.2.3. (Ersts 2012) and log transformed for analysis (Rousset 1997). The significance of this relationship was tested using a Mantel test with 10,000 randomizations in the Isolation by Distance Web Service v. 3.23 (Jensen et al. 2005).

#### Host fungus community diversity

Host fungus community diversity in each of the five plots was quantified with the Shannon–Wiener equitability index (also known as Pielou's *J*; Pielou 1969), which measures the relative abundance of species that comprise a community. The index (*J′*) is given by


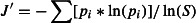


where *p*_*i*_ is the frequency of the *i*^th^ species, and *S* is the total number of species in the community. The numerator is the Shannon diversity index, *H′*; the denominator is equivalent to *H′*_*max*_, the maximum value that *H′* can take, given *S* species. As a result, *J′* ranges from zero to one, with zero corresponding to a community with a single species, and one to a community where all species are equally abundant. We chose a measure of evenness instead of richness (the number of species in the community) in order to distinguish between host communities in which all host species are present, but in very different proportions.

To calculate *J′* for each plot, we counted all logs in the plot that were infected by the three host fungi. Because we intended this index to reflect the community of available host fungi, we excluded logs that did not exhibit any evidence of *B. cornutus* (i.e., neither adults nor eggs were present) or on which all brackets were in an advanced state of decay. Logs that were coinfected by two of the host fungi (20.9% of all logs) were counted twice, once for each fungus species. All three fungi were present in all five plots.

We performed an AMOVA separately for each plot in order to test the hypothesis that community diversity affects population differentiation. This analysis calculated *F*_ST_ in each plot. We assessed the relationship between the Shannon–Wiener equitability index and these *F*_ST_ values using Spearman's rank correlation. Each observation in this data set was one of the five study plots. This analysis was performed in R 2.10.1 (R Development Core Team 2010).

## Results

We genotyped a total of 596 individuals from 63 logs, including 508 individuals from 43 logs at the main study site at Mountain Lake Biological Station. In this sample, 173 beetles were collected from *F. fomentarius*, 56 from *G. applanatum*, 161 from *G. tsugae*, and 118 from coinfected logs.

**Figure 3:**
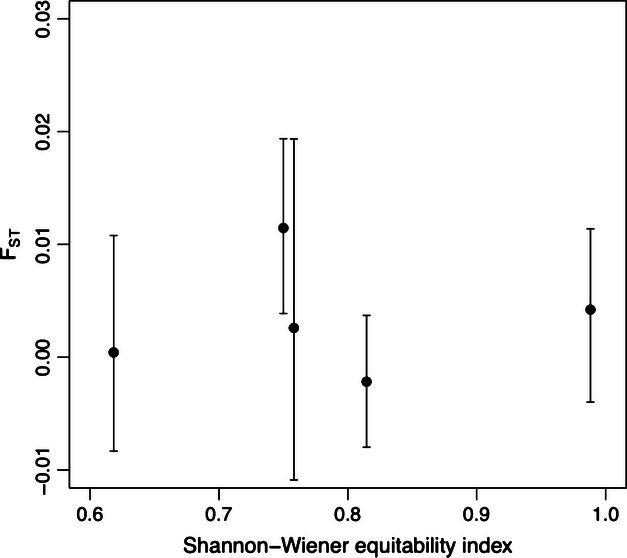
The relationship between community diversity (Shannon–Wiener equitability index) and population differentiation (*F*_ST_). Each point corresponds to one of the five study plots. Error bars represent 95% confidence intervals obtained by bootstrapping over loci. This relationship was not significant (Spearman's ρ = 0.0, *N* = 5, *P* = 1.0).

We found no significant relationship between host community diversity and *F*_ST_ across the five study plots (Spearman's ρ = 0.0, *N* = 5, *P* = 1.0; Fig. 3). We also found that the three host fungi did not significantly contribute to population structure (*F*_CT_ = 0.001, *P* = 0.36; Table 1). This pattern held across all nine microsatellite loci. Overall, the level of population differentiation was extremely low (*F*_ST_ = 0.0021 [95% confidence interval: −0.0003 to 0.0049], *P* = 0.31; Table 1), a result that is inconsistent with a previously published study that estimated a moderate level of population structure on the same mountain along with one of the same drainages (*F*_ST_ = 0.017; Whitlock 1992). When *F*_ST_ is calculated separately for each locus, only one locus approaches the level of population structure measured by Whitlock (Boco_128: *F*_ST_ = 0.012), but this value was not significant. However, we did detect a significant inbreeding coefficient (*F*_IS_ = 0.02; Table 1).

**Table 1:** Hierarchical analysis of molecular variance (AMOVA), partitioning total genetic variance into the following components: within logs (*F*_IS_), within fungi (*F*_SC_), among fungi (*F*_CT_), and among all logs (*F*_ST_)

Locus	*F*_IS_ within logs	*P*	*F*_SC_ within fungi	*P*	*F*_CT_ among fungi	*P*	*F*_ST_ among logs	*P*
Boco_049	0.0077	0.41	0.0002	0.47	0.0020	0.19	0.0022	0.4
Boco_030	0.0189	0.28	−0.0008	0.60	0.0025	0.11	0.0018	0.46
Boco_007	0.0343	0.14	−0.0055	0.85	0.0043	0.04	−0.0012	0.71
Boco_006	0.0399	0.18	−0.0005	0.58	−0.0020	0.71	−0.0025	0.63
Boco_061	0.0358	0.19	0.0009	0.53	−0.0023	0.80	−0.0014	0.6
Boco_065	0.0372	0.06	0.0010	0.50	−0.0016	0.83	−0.0006	0.63
Boco_034	−0.0458	0.93	0.0019	0.28	0.0007	0.28	0.0026	0.23
Boco_128	0.0421	0.16	0.0151	0.05	−0.0031	0.65	0.0120	0.1
Boco_084	0.0253	0.15	0.0093	0.10	−0.0016	0.70	0.0077	0.1
All loci	**0.0209** (0.0013 to 0.0345)	0.02^*^	0.0020 (−0.0024 to 0.0047)	0.35	0.0001 (−0.0014 to 0.0018)	0.36	0.0021 (−0.0003 to 0.0049)	0.31

See Methods for a detailed explanation of the four *F*-statistics. Results are presented for each of the nine microsatellite loci, as well as the weighted average over all loci. 95% confidence intervals for the multilocus *F*-statistics are reported in parentheses and were obtained by bootstrapping over loci. Significant values (*P* < 0.05) are indicated in bold, with an asterisk.

When the data were analyzed separately for the two sexes, the host fungi still did not affect population structure within each sex (Table 2). Moreover, there was no evidence of sex-biased dispersal irrespective of fungus species, as *F*_ST_ was not significantly different from zero in either sex. We detected a significant inbreeding coefficient in males but not in females.

**Table 2:** Hierarchical analysis of molecular variance (AMOVA) performed separately for the two sexes, partitioning total genetic variance into the following components: within logs (*F*_IS_), within fungi (*F*_SC_), among fungi (*F*_CT_), and among all logs (*F*_ST_)

	*F*_IS_ (within logs)	*P*	*F*_SC_ (within fungi)	*P*	*F*_CT_ (among fungi)	*P*	*F*_ST_ (among logs)	*P*
Females	−0.0016 (−0.0392 to 0.0262)	0.52	−0.0005 (−0.0086 to 0.0078)	0.54	−0.0027 (−0.0051 to 0.0004)	0.96	−0.0033 (−0.0108 to 0.0045)	0.72
Males	**0.0396** (0.0101 to 0.0709)	0.02^*^	−0.0021 (−0.0137 to 0.0110)	0.63	0.0028 (−0.0043 to 0.0100)	0.16	0.0033 (−0.0054 to 0.0152)	0.49

See Methods for a detailed explanation of the four *F*-statistics. 95% confidence intervals are reported in parentheses and were obtained by bootstrapping over loci. Significant values (*P* < 0.05) are indicated in bold, with an asterisk.

**Figure 4:**
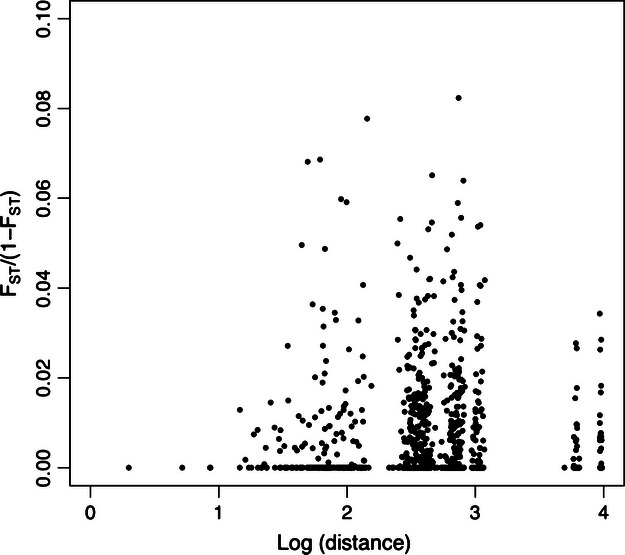
The relationship between Slatkin's linearized *F*_ST_ (D = *F*_ST_/(1 − *F*_ST_)) and the logarithm of geographic distance. This relationship was not significant (Mantel test; *r* = −0.016, *P* = 0.51).

The isolation-by-distance analysis did not reveal a significant relationship between genetic distance and geographic distance (Mantel test; r = −0.016, *P* = 0.51; Fig. 4). Populations separated by nine kilometers were not more likely to be genetically distinct than those separated by a few meters.

## Discussion

We found no evidence that the host community contributes to population structure in this *B. cornutus* metapopulation. However, we also found that population structure appears to be unrelated to the host fungi in this species, indicating that host fungi do not constitute a substantial barrier to gene flow among *B. cornutus* subpopulations. In contrast to an earlier study, we found no evidence for population structure, even between populations separated by nearly ten kilometers and located on another mountain.

The lack of a relationship between host community diversity and population differentiation is consistent with the fact that the host fungi do not seem to pose a barrier to gene flow in *B. cornutus*. There are a number of reasons that may account for the absence of host-associated differentiation in this system. First, the three hosts may not be characterized by divergent selection, allowing migrants to move freely among habitats. Two of the three hosts are congeners, so adaptation to the chemical profile of one host may have positive effects on performance on the other. Moreover, in a survey of bracket-feeding insects, Jonsell and Nordlander (2004) found that *F. fomentarius* and *G. applanatum* were the most commonly used pair of hosts; insects that fed on one were likely to use the other as well. As a result, tradeoffs in performance across the three host fungi may be relatively minor, unlike in species that exhibit pronounced local adaptation to a host (e.g., aphids, Ferrari et al. 2008).

An alternative possibility is that although *B. cornutus* populations on different hosts experience divergent selection, frequent migration on the microgeographic scale of this study prevents the emergence of host-associated population structure. One aspect of the ecology of the system that may promote migration among fungi is intra- or interannual variation in relative abundance of the hosts. One of the host fungi (*G. tsugae*) produces brackets on an annual basis, so it tends to be less abundant in the spring and becomes more common as the summer progresses. As it becomes a more abundant resource, it likely absorbs migrants from the other two host fungi, erasing genetic differentiation between populations on different fungi. Interannual variation in the relative abundance of the three host fungi would have a similar effect. Such temporal heterogeneity in the host fungus community could generate selection for ecological generalists who are able to exploit whichever fungus is most abundant during a given month or year (Kassen 2002).

In contrast to a previous mark-recapture study of dispersal in *B. cornutus* (Schwarz 2006), there is no genetic evidence from our study that the fungus on which they originate affects *B. cornutus* dispersal. Furthermore, because the contribution of host fungus to genetic structure does not differ between males and females, there is no evidence for habitat choice in either sex. This result is somewhat surprising because ovipositing females often exhibit habitat choice for a particular host, especially when their offspring rely on the host throughout development (Resetarits 1996), as is the case in *B. cornutus*. Females of some species are more sensitive to chemical volatiles diagnostic of a particular host species, probably for this reason (Faldt et al. 1999). We did find a significant positive inbreeding coefficient in the complete data set (*F*_IS_ = 0.02; Table 1), but only in males when the two sexes were analyzed separately (Table 2). The biological interpretation and significance of this result remain unclear. It may be indicative of kin structuring within subpopulations, although it is not immediately evident why such an effect should be stronger in males.

It may be that the costs of habitat choice in this system outweigh the benefits. This is more likely to be true if habitat choice is energetically expensive, either because choosy individuals migrate longer distances before locating a log infected with their preferred fungus, or because the sensory apparatus required to discriminate among habitats is costly to maintain. In the latter case, constraint may play a role in preventing the development of host-associated population structure in *B. cornutus*. Bracket fungi emit cocktails of volatile compounds that dispersing insects exploit to locate infected logs (Faldt et al. 1999). Closely related bracket fungi differ in the chemical composition of these volatiles, and some, but not all, insects are able to discriminate between hosts on this basis (Faldt et al. 1999). A previous lab-based experiment demonstrated that *B. cornutus* prefer *G. applanatum*, suggesting that *B. cornutus* can discriminate among the hosts (Heatwole and Heatwole 1968). It is possible that even though they are capable of distinguishing hosts, the cost of habitat choice in the field, imposed by dispersal, outweighs the host preference exhibited in the laboratory.

Many canonical examples of microhabitat specialization and concomitant patterns of population genetic structure are found in host-associated insects (Feder et al. 1994; Mopper 1996; Via et al. 2000; Stireman et al. 2005). However, host-associated differentiation in insects is certainly not ubiquitous; there are a number of species in which the host resource has no measureable impact on patterns of population differentiation (Van Zandt and Mopper 1998; Stireman et al. 2005; Jourdie et al. 2010; Kohnen et al. 2011). Certain aspects of insect life history seem to predispose some species to host specialization, which is disproportionately common in endophagous and parthenogenetic species (Van Zandt and Mopper 1998; Stireman et al. 2005). Endophagous species like gall makers come into especially close contact with the host defenses, generating stronger selection for specialization; parthenogenesis results in a tighter association between insect and host genotypes, accelerating divergence among hosts.

Yet exceptions to this pattern exist even among gall-makers and parthenogens. There is no signature of host-associated differentiation in a rose gall wasp (*Diplolepis rosae*), a species in which *Wolbachia* infection results in parthenogenesis (Kohnen et al. 2011). Population genetic structure in bruchid beetles, the larvae of which consume the developing seeds of legumes, is not affected by the host bean species (Restoux et al. 2010). In a study of two insects associated with hosts in the genus *Pinus* but differing in life-history traits such as endophagy, Kerdelhué et al. (2006) found no effect of host plant on genetic structure in either species. Finally, the results of this study demonstrate that host fungi are not associated with population differentiation in *B. cornutus*, an endophagous beetle. This body of results underscores the fact that host-associated population differentiation is a complex phenomenon that can be difficult to predict (Kohnen et al. 2011). A diversity of ecological factors – including but not limited to the grain of environmental heterogeneity, the magnitude of differences among hosts, insect life histories, and the frequency of migration among hosts – govern the extent to which gene flow between populations on different hosts is constrained.

A comparison between this study and a previously published study in the same geographic area (Whitlock 1992) suggests that population structure in *B. cornutus* may be temporally labile. We found little or no population structure in our study population: beetles on logs separated by nearly ten kilometers were not more genetically distinct than those on neighboring logs (Fig. 4). However, Whitlock (1992) reported moderate differentiation (*F*_ST_ = 0.017) on the same mountain. It is worth noting that Whitlock (1992) analyzed allozyme variation, whereas our present study examined microsatellites. However, these two different marker types typically produce similar estimates of population differentiation, especially after outlier allozymes are excluded (Estoup et al. 1998; Dhuyvetter et al. 2004; Roberts and Weeks 2011). Although it is difficult to speculate about the mechanism responsible for driving the change in population structure in the past two decades, one possibility is that the age structure of populations in the study area has changed substantially. Whitlock's (1992) estimate of moderate population differentiation was primarily driven by the younger (more recently colonized) logs in his sample. He concluded that younger populations were more genetically differentiated than older ones due to founder effects, so a decline in the proportion of young populations could result in a corresponding decrease in *F*_ST_. The magnitude of the reduction in *F*_ST_ between younger and older populations differed between the two sites in Whitlock's study; although there was no detectable genetic differentiation among older populations at one site, at the site corresponding to the location of this study *F*_ST_ among older populations was 0.015. This suggests that even a substantial change in age structure may not be sufficient to account for the difference between Whitlock's (1992) study and this study.

An alternative hypothesis for disappearance of microgeographic population structure in *B. cornutus* involves the dynamic nature of the host fungus community on a longer time scale. Temporal variation in host community structure could have erased resource-associated population differentiation, because a decline in the frequency of one host would force beetles previously associated with it to colonize the other two. It is possible that host fungus community diversity has changed substantially on Salt Pond Mountain in the two decades separating this study from Whitlock's, due to the invasion of the hemlock woolly adelgid (*Adelges tsugae*). Widespread eastern hemlock (*Tsuga canadensis*) decline, driven by the invasion of hemlock woolly adelgid, may have led to an increase in the frequency of *G. tsugae* – a hemlock specialist. Hemlock woolly adelgid is a sap-sucking insect that feeds at the base of hemlock needles, killing the tree (Young et al. 1995; Stadler et al. 2006; Evans and Gregoire 2007). Its impact on tree communities – and corresponding effect on their associated fungi – is likely most pronounced in riparian forests in the southern Appalachians, such as our study site, where hemlock is the dominant tree species (Webster et al. 2012). Both standing dead hemlock snags and adelgid-infected live hemlocks are common at our study site.

An invasive from Asia, hemlock woolly adelgid was first recorded in North America in 1951 near Richmond, VA, and since has spread into the Appalachian Mountains south to Georgia and north to Maine (Fitzpatrick et al. 2012). The first hemlock woolly adelgid infestation in Giles County, VA (the location of this study) was recorded in the early 1990s (Fitzpatrick et al. 2012; US Forest Service data available online), shortly after Whitlock's data were collected in 1988. As a result, dead hemlocks are probably much more abundant in the study site than they were in the late 1980s, and may have driven a corresponding increase in the frequency of *G. tsugae* brackets. Whether exploitation of this newly abundant resource may have erased population genetic structure in *B. cornutus* that Whitlock (1992) observed remains to be tested.

This study accords with a growing body of evidence suggesting that population genetic structure can be surprisingly dynamic (Charbonnel et al. 2002; Heath et al. 2002; Østergaard et al. 2003; Apodaca et al. 2013). In some cases, temporal changes in population structure are associated with catastrophic events such as hurricanes (e.g., sailfin mollies; Apodaca et al. 2013), whereas in others, population structure varies among years in the absence of obvious large-scale ecological drivers (e.g., steelhead trout; Heath et al. 2002). Many more temporally replicated estimates of population structure are necessary to assess its lability in the short and long term, and to identify the environmental factors associated with the restructuring of genetic variance in natural populations.

Finally, the degree to which genetic structure reflects the environment may differ across different regions of the genome, particularly in the early stages of local adaptation (Via 2009). Under divergent selection in different habitats, loci that are associated with resource utilization exhibit pronounced divergence relative to neutral markers like microsatellites. This is the principle underlying methods such as *Q*_ST_-*F*_ST_ comparisons: Regions associated with ecologically important quantitative traits may show high levels of divergence not reflected in neutral genetic variation (Scheffer and Hawthorne 2007). Thus, the geographic distribution of genetic variance at non-neutral loci may be very different from that at neutral loci. For example, in a study of moor frogs, a wetland-breeding amphibian, Richter-Boix et al. (2011) found that although population structure at most microsatellite loci was unaffected by environmental differences among wetlands, one locus exhibited habitat-associated structure. The authors inferred that this single locus was likely experiencing selection for local adaptation not reflected in the neutral loci. The *B. cornutus* genome could be a mosaic in which loci that affect habitat use are characterized by host-associated genetic structure, while neutral loci are not. However, all nine microsatellite loci included in this study yielded similar estimates of population structure, providing little evidence for this interpretation.

Although this study did not find a relationship between host community diversity and population differentiation, such a relationship may exist in populations that do exhibit habitat-associated population structure, unlike *B. cornutus*. Because habitat-associated population structure is common in other systems, the most robust test of our hypothesis would be conducted in a species in which gene flow among environments is known to be restricted (e.g., Hoekstra et al. 2004; Nosil and Crespi 2004; Stireman et al. 2005). Future studies in species that are characterized by pronounced habitat-associated genetic differentiation are necessary to elucidate the effect of landscape-level habitat diversity on patterns of population genetic structure.
